# Pentacam HR Indices Variation in Normal Corneas with Different Corneal Thickness

**DOI:** 10.1155/2018/9328120

**Published:** 2018-11-08

**Authors:** Maged Maher Salib Roshdy, Sherine Shafik Wahba, Rania Serag Elkitkat, Nermine Said Madkour, Ramy Riad Fikry

**Affiliations:** ^1^Ophthalmology Department, Ain Shams University, Al Watany Eye Hospital and Watany Research and Development Center (WRDC), Cairo, Egypt; ^2^Ophthalmology Department, Al Watany Eye Hospital and Watany Research and Development Center (WRDC), Cairo, Egypt; ^3^Ophthalmology Department, Cairo University, Al Watany Eye Hospital and Watany Research and Development Center (WRDC), Cairo, Egypt

## Abstract

**Purpose:**

To evaluate the effect of variable corneal thickness on Pentacam HR diagnostic indices in normal corneas.

**Methods:**

Retrospective study was conducted at Al Watany Eye Hospital, Cairo, Egypt. Consecutive 160 eyes of young myopic subjects without KC were evaluated using Pentacam HR (WaveLight Allegro Oculyzer II, Erlangen, Germany). The elevation- and thickness-based indices were recorded. Enrolled corneas were categorized into three groups according to TCT quartiles; group 1 (39 eyes) included corneas with TCT <523 *µ*m, group 2 (81 eyes) with TCT between 523 and 564 *µ*m, while group 3 (40 eyes) enrolled TCT >564 *µ*m. The possible effect of pachymetry on Pentacam HR indices was assessed using partial correlation tests.

**Results:**

In normal corneas, back elevation from best fit sphere (BE from BFS) and that from best fit toric ellipsoid (BFTE) were the elevation indices that showed statistically significant differences among groups (*P*=0.013 and 0.019, respectively). Regarding pachymetric indices, maximum pachymetry progression index (PPI max) showed statistical significance (*P*=0.001). Partial correlations, after excluding age and refractive error effects, showed that TCT was correlated with BE from BFS, BE from BFTE, and PPI max (*P*=0.001, 0.001,0.002, respectively).

**Conclusions:**

Some Pentacam HR indices varied with different corneal thickness in normal corneas. This necessitates inclusion of pachymetric subgroups in the normative database. The use of the more robust indices (average pachymetry progression index and front elevations) is recommended in relatively thin or thick corneas.

## 1. Introduction

In the past few decades, corneal evaluation prior to refractive surgery was performed using topography systems with “Placido-based” technology, which was considered inaccurate by some authors [1–3], as it lacks the assessment of the posterior corneal surface and corneal pachymetry. These important data were later detected using “Elevation-based” corneal imaging techniques, with the Pentacam HR (OCULUS GmbH, Wetzlar, Germany) being one of the most popular devices [4].

Pentacam HR calculates a range of indices from curvature, elevation, and pachymetric data [5]. Thinnest corneal thickness (TCT) and other pachymetric indices have been widely discussed in the literature and are frequently considered as important KC screening parameters [6], as well as major risk factors for postoperative ectasia development [7, 8]. Safe values for residual stromal bed thickness following laser in situ keratomileusis (LASIK) [9, 10], and the acceptable percentage of tissue altered [11, 12] has been previously highlighted. All these studies reflect a robust role of corneal thickness as a determinant of corneal properties and an ectasia screening parameter [13–15].

Recent corneal studies aroused an important issue that deserves proper analysis. Some factors can be correlated with various Pentacam indices, and variations in these factors can significantly alter indices values and, hence, alter their interpretation. The effects of age [16, 17] and refractive errors [18] on Pentacam detection indices have been highlighted. This led us to plot this study design, aiming to evaluate the possible existence of correlations between corneal thickness, as a robust factor by itself, and other elevation and pachymetry-based indices using Pentacam HR, and the consequent need to adjust the normative values accordingly.

## 2. Methods

This is a retrospective study at Al Watany Eye Hospital, which enrolled myopic eyes of 160 consecutive subjects having normal, nonectatic corneas. All recruited participants were seeking refractive surgical correction. We excluded candidates with any detected corneal pathology, contact lens wear within the previous two weeks, narrow palpebral fissure precluding proper scanning, or previous ocular surgery. The study adhered to the tenets of the Declaration of Helsinki and was approved by the Watany Research and Development Center (WRDC) Institutional Review Board.

Both eyes for each patient were evaluated using Pentacam HR, branded as Allegro Oculyzer II (WaveLight GmbH, Erlangen, Germany). Each eye was scanned according to the recommendations of the device manual (at least thrice), and the most reliable scan was chosen regarding the largest analysed area, valid data percent, and good alignment. The eye with the most reliable scan was then selected for analysis using software version 120r20.

The investigated indices included the following:

- Elevation-based indices (float, all obtained from 8 mm zone)
  - Front elevation of the thinnest point (FE) from best fit sphere (BFS)
  - Back elevation of the thinnest point (BE) from BFS
  - FE from best fit toric ellipsoid (BFTE)
  - BE from BFTE
- Pachymetry-based indices
  - TCT
  - Average and maximum corneal pachymetry progression indices (PPI avg and PPI max, respectively)
  - Average and maximum Ambrosio's relational thickness indices (ART avg and ART max, respectively)

To keep aside the bias that could arise from the effect of age [17] and manifest refractive spherical equivalent (SE) and mean K readings (Km), we included them in the evaluation.

The corneas enrolled in the study were categorized into three groups, according to TCT quartiles of this sample; each quartile comprises 25% of corneas.

### 2.1. Statistical Analysis

Data were collected and verified, and the compound indices were calculated. The following tests were performed using IBM SPSS Statistics (v22; Armonk, NY, USA): calculation of the mean, standard deviation (SD), one-way analysis of variance (ANOVA), Pearson and its nonparametric equivalent Spearman correlation coefficients, and partial correlation coefficients controlling for SE, Km, and age. Values were considered statistically significant if the *P* value was less than 0.05.

## 3. Results

Subjects' mean (±SD) age was 27.7 ± 6.3 years (ranging from 18 to 45). SE had a mean of 4.96 ± 2.81 diopters (D) (ranging from −0.625 to −12.5), including simple myopia and/or myopic astigmatism. The mean TCT was 543.3 ± 32.4 *µ*m (ranging from 463 to 648 *µ*m).

Group 1 (39 eyes) included thin corneas of TCT 463 to 522 *µ*m (1st quartile), group 2 comprised corneas of 2nd quartile (523 to 540 *µ*m, 41 eyes) and 3rd quartile (541 to 564 *µ*m, 40 eyes), while group 3 (40 eyes) enrolled thick corneas of TCT between 565 and 648 *µ*m (4th quartile).

Regarding the distribution of subjects' age, SE, and Km, they showed no statistically significant differences among groups, and consequently no bias can arise from them (Table 1).

The mean, SD, together with the suggested alarming values (based on mean ±2 and 3 SD) of all studied indices in the 3 groups are shown in Table 2. For ART avg and ART max, the alarming values were those less than the mean −2 or −3 SD, while for other indices, the alarming values were those greater than the mean +2 or +3 SD.

### 3.1. Elevation-Based Indices

BE from BFS was significantly different among groups (*P*=0.013). The same was found regarding the BE from BFTE (*P* = 0.019). On the contrary, FE from BFS and BFTE did not show any statistically significant differences.

The post hoc test showed that thick corneas “group 3” had higher statistically significant BE from BFS and BFTE than thin corneas “group 1” (*P*=0.02 and 0.005, respectively) and than average thickness corneas “group 2” (*P*=0.018 and 0.009, respectively).

### 3.2. Pachymetry-Based Indices

One-way ANOVA test detected a statistically significant difference among groups regarding PPI max (*P*=0.001), ART avg (*P* < 0.001), and ART max (*P* < 0.001), while PPI avg showed no statistical significance (*P*=0.055).

The post hoc test showed that thinner corneas “group 1” had PPI max greater than thicker corneas “group 3” (*P*=0.001). On the contrary, ART avg and ART max increased from group to group with the increase in thickness (*P* < 0.001 in all).

### 3.3. Partial Correlations

After exclusion of the age, refraction, and Km effects, partial correlation tests demonstrated that TCT was correlated with BE from BFS (*P*=0.001), BE from BFTE (*P*=0.001), and PPI max (*P*=0.002). On the contrary, TCT was not correlated with FE from BFS (*P*=0.615), FE from BFTE (*P*=0.626), and PPI avg (*P*=0.177).

## 4. Discussion

The frequent use of Pentacam nowadays for meticulous prerefractive surgery assessment, aiming at minimizing the risk of post-LASIK ectasia, has attracted the attention of researchers in the past decade, owing to the significant negative impact of post-LASIK ectasia on visual capacity, even in the early stages of the disease [19]. This interest has opened the field for various studies, that enriched literature with data regarding the accuracy of various ectasia detection indices [20, 21, 22]. Many studies detected a higher accuracy for tomographic (elevation- and pachymetry-based) rather than topographic (curvature-based) indices in ectasia diagnosis [22, 23]. Hence, we focused, in this study, on evaluating any possible correlations of variable corneal thickness profiles with various tomographic rather than topographic indices.

We categorized our cohort of normal corneas into groups according to TCT quartiles of this sample, and also the average TCT is near the values published in the literature that evaluated TCT measurement using Pentacam [24–27]. The results showed TCT significant impact on some indices and insignificant impact on others. Regarding elevation-based indices, BE from BFS and from BFTE were different among the 3 studied groups, with thicker corneas (group 3) showing significantly higher values compared with the other 2 groups, whereas FE from BFS and BFTE did not show any statistical significance. As for pachymetry-based indices, PPI max was the index that showed statistically significant difference among groups, where thinner corneas (group 1) had significantly higher values compared with the other 2 groups. On the contrary, PPI avg was statistically invariable. Hence, relying on FE from BFS and BFTE in thicker corneas, PPI avg for thinner corneas is recommended.

These findings could be, in our view, because of two possibilities. Firstly, thick corneas may be morphologically different from thinner ones [18] and therefore have a different relationship between central and peripheral zones, causing different PPI, BE from BFS, and BFTE.

Secondly, corneas with all thickness ranges may have similar morphology, but are differently assessed by the imaging device if corneal thickness values are above or below those of the adjusted normative database. This issue was previously detected in the Orbscan slit-scanning device [28] and may also exist in Scheimpflug devices, as the posterior surface measurements can be theoretically affected by the fact that Scheimpflug cameras capture images of inner structures through the outer cornea.

Further studies assessing the same indices using different imaging technologies, as the optical coherence tomography, may elucidate which of these two hypotheses is more plausible.

We presented the 2 and 3 SD values for the evaluated indices as previously suggested [18]. These modified normative values can be relied upon according to different TCT subgroups.

This study is complementing other studies that have recently discussed a possible effect of some factors on elevation and pachymetry-based indices. For instance, the age factor has been previously discussed, evaluating its effect on topometric indices, FE and BE [16], and on other KC detection indices [17]. Moreover, the possible impact of refractive errors on the accuracy of tomographic KC detection indices (FE, BE, TCT, and corneal thickness at apex) has been evaluated by Kim and coworkers [18]. These studies recommended changing the normative database with variations in age and refractive errors, respectively. In newer Pentacam HR software, refractive errors grouping is now incorporated.

To the best of our knowledge, this is the first study to correlate TCT with various tomographic indices. Moreover, the study suggested two possible hypotheses. Validation of our results with further studies, including larger cohorts, together with evaluating the possible existence of such correlations between pachymetry and other indices using other Scheimpflug devices rather than Pentacam HR, is highly recommended.

We assume that our study has an important clinical relevance, as we highlight the possible fallacies of relying on all Pentacam indices using current normative database, without taking into consideration the pachymetric subgrouping of cohorts. These results highlight the importance of including pachymetric subgroups in Pentacam future software versions, as there is no such pachymetry subgrouping in the current software.

## 5. Conclusion

Some Pentacam HR indices may be affected by corneal pachymetry. This necessitates the inclusion of pachymetric subgroups in Pentacam normative database. Up till then, the use of the more robust FE and PPI avg is recommended in relatively thick or thin corneas.

## Figures and Tables

**Table 1:** Age, refractive spherical equivalent (SE), and mean keratometry (Km) among the studied groups.

	Group 1		Group 2		Group 3		ANOVA
	Mean	SD	Mean	SD	Mean	SD	*P* value
Age	29.0	7.2	27.5	5.6	26.9	6.7	0.290
SE	–5.23	3.10	–5.10	2.55	–4.43	3.03	0.374
Km	43.74	1.47	43.58	1.48	44.01	1.32	0.305

SD = standard deviation.

**Table 2:** The mean, standard deviation (SD), and the suggested cutoff values (2 and 3 SD from the mean) of different parameters in the three groups.

Index	Group 1				Group 2				Group 3			
	Mean	SD	2 SD limit	3 SD limit	Mean	SD	2 SD limit	3 SD limit	Mean	SD	2 SD limit	3 SD limit
PPI avg	0.980	0.117	1.214	1.332	0.926	0.102	1.130	1.233	0.930	0.142	1.214	1.355
PPI max	1.244	0.168	1.580	1.748	1.132	0.143	1.418	1.561	1.120	0.187	1.494	1.681
ART avg	522.3	66.4	389.5	323.1	591.6	68.1	455.4	387.3	642.8	91.3	460.3	369.1
ART max	413.4	61.9	289.6	227.7	485.8	64.2	357.3	293.1	536.7	89.8	357.1	267.3
FE from BFS	3.4	1.3	6.0	7.3	3.1	1.5	6.0	7.4	3.2	1.8	6.7	8.5
BE from BFS	4.7	3.6	11.9	15.5	4.6	4.0	12.7	16.7	6.9	4.4	15.6	20.0
FE from BFTE	0.2	1.0	2.1	3.1	0.1	1.0	2.0	3.0	0.1	0.9	2.0	2.9
BE from BFTE	0.3	2.6	5.5	8.1	0.4	2.4	5.3	7.7	1.7	2.8	7.3	10.2

ART avg = average Ambrosio's relational thickness index, ART max = maximum Ambrosio's relational thickness index, PPI avg = average pachymetry progression index, PPI max = maximum pachymetry progression index, FE = front elevation of the thinnest point, BFS = best fit sphere, BE = back elevation of the thinnest point, BFTE = best fit toric ellipsoid, and SD= standard deviation.

## Data Availability

The data used to support the findings of this study are included within the article.
